# Implementing a Primary Care Referral Pathway for Further Investigations and Management of Fatty Liver Disease (FLD) in the Absence of Fibrosis Identified in Patients Who Have Undergone a Fibroscan Within the Belfast Addictions Service

**DOI:** 10.1192/bjo.2024.352

**Published:** 2024-08-01

**Authors:** Zuhayr Booley

**Affiliations:** Addictions Service, Belfast, United Kingdom

## Abstract

**Aims:**

Alcohol misuse presents a major health concern in Northern Ireland with a 50% rise in alcohol-specific deaths in the past 10 years^1^. Excessive alcohol use may lead to fat deposition within the liver and risks progressive liver disease secondary to fibrosis and/or inflammation. The aim is to extend upon an existent Hepatology referral pathway for patients with alcohol misuse and liver fibrosis on Fibroscan; to include onward referral to primary care for investigations of patients shown to have FLD in absence of fibrosis and facilitate early identification and intervention of associated metabolic syndromes. There was previously no referral mechanism for screening for metabolic syndromes such as diabetes, hypertension and hypercholesterolemia for these patients.

**Methods:**

Case records were reviewed for all patients offered a Fibroscan through the Belfast Addictions service. Patients identified with evidence of steatosis on Fibroscan without fibrosis/cirrhosis i.e. liver stiffness score < 8Kpa and controlled attenuation score (CAP) > 248, would trigger an onward referral to primary care for further investigation and management. A letter was sent notifying the patients’ registered GP of the Fibroscan result and NICE recommendations for follow up liver function testing, HbA1c, lipid profile and Q-risk scoring for consideration of lipid lowering medication. A review of patients’ electronic care record (ECR) 2 months following the dispatch of letters was conducted to identify those patients who received further investigations.

**Results:**

286 Fibroscans were conducted in the Belfast Addictions Service in 2023. Alcohol misuse was the indication for 92% of these scans with 32% identified as having evidence of fatty liver disease without fibrosis. This prompted onward referral for primary care follow up and letters were sent out to GPs from November 2023. Review of ECR 2 months post-intervention revealed of the 7 letters sent out in November, 57% (4) had follow up bloods and 75% (3) of those were shown to be deranged. Data collection is ongoing and will be complete by date of congress.

**Conclusion:**

32% of the patients who had a Fibroscan in the Belfast Addictions service in 2023 had evidence of fatty liver disease without cirrhosis. Initial data shows a positive change in clinical practice and patient care, and builds upon the existing hepatology referral pathway.

